# Vegetation phenology and nest survival: Diagnosing heterogeneous effects through time

**DOI:** 10.1002/ece3.4906

**Published:** 2019-01-21

**Authors:** Kevin M. Ringelman, Cassandra G. Skaggs

**Affiliations:** ^1^ School of Renewable Natural Resources Louisiana State University AgCenter Baton Rouge Los Angeles

**Keywords:** bias, duck, grassland, mallard, prairie, Robel, waterfowl

## Abstract

Birds should select nest sites that minimize predation risk, but understanding the influence of vegetation on nest survival has proven problematic. Specifically, the common practice of measuring vegetation on nest fate date can overestimate its effect on nest survival, simply because vegetation at hatched nests grows for a longer period of time than vegetation at nests that were depredated. Here, we sampled the literature to determine the prevalence of this bias in studies of duck breeding ecology. We then used survival data collected from ~2,800 duck nests to empirically evaluate evidence of bias in four different vegetation metrics: vegetation density measured when the nest was found, density when the nest was fated, and date‐corrected regression residuals of these two. We also diagnosed the magnitude of vegetation effects on nest survival by restricting our analysis to only nests which were fated contemporaneously (thereby removing potential bias in the timing of measurement). Finally, we examined whether systematic phenological differences exist between vegetation at hatched and depredated nests that have the potential to further obfuscate the relationship between vegetation and nest survival. We found evidence for a true‐positive effect of vegetation density on nest survival that appeared to be inflated when using raw vegetation measurements collected at fate date. However, taken in combination with the literature review, our results suggest that the majority of duck nesting studies have evaluated the role of vegetation on nest survival using a relatively less biased metric—vegetation density when the nest was found. Finally, we found that over the course of a nesting attempt, vegetation increased in density at successful nests, but decreased in density at depredated nests. As a consequence, duck researchers using vegetation data collected when the nest was found may actually be underestimating the magnitude of the effect. This seasonal change potentially points to an important new metric for understanding predation risk, but further experimental research is required to fully eliminate potential biases in the timing of vegetation measurements.

## 1 INTRODUCTION

**Figure 1:**
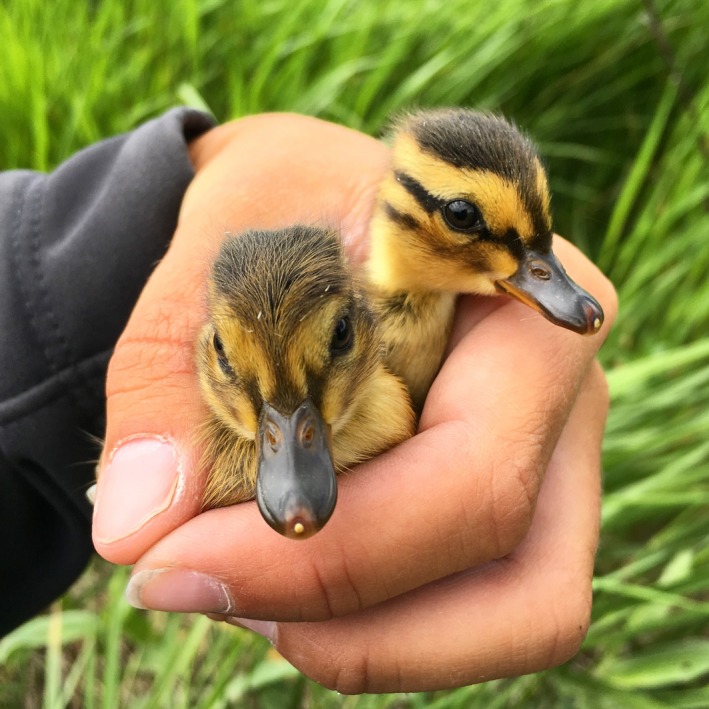
Mallard (*Anas platyrhynchos*) duckling

Predation is the primary cause of nest failure in most bird species (Ricklefs, 1969), and nest survival (Figure 1) can be a major limiting factor to population growth (Hoekman, Mills, Howerter, Devries, & Ball, 2002; Wisdom & Mills, 1997). Thus, it comes as no surprise that birds have evolved a remarkable array of behavioral and life‐history strategies to ameliorate the risk of nest predation (reviewed in Lima, 2009). Perhaps chief among them is adaptively choosing safe nest sites with vegetative characteristics that conceal nests from potential predators (Martin, 1993), though the degree to which birds actually manage this is a perennial source of debate among ornithologists (Chalfoun & Schmidt, 2012; Clark & Shutler, 1999). Nevertheless, understanding the habitat needs of breeding birds is of paramount importance in the conservation and management of these species (Lebbin, Parr, & Fenwick, 2010). Researchers therefore dedicate substantial resources to measuring nesting habitat, ranging from nest microclimate (Marzluff, 1988) to landscape‐level patterns of fragmentation (Laurance et al., 2011).

Vegetation is heterogeneous in space and time, especially during the spring growing season when most birds are nesting. Therefore, understanding the influence of vegetation on key aspects of breeding ecology (e.g., habitat selection, nest survival, brood survival) hinges on measuring vegetation at a time that is appropriate for the research question of interest (Borgmann & Conway, 2015; Burhans & Thompson, 2001). This is not always straightforward. Consider the relationship between vegetation density and the risk of nest predation: measuring nest vegetation at the end of the breeding season could obscure that correlation, because conditions at the end of the season may not reflect vegetation density at the time the nest was actually discovered by a predator (Borgmann & Conway, 2015; Burhans & Thompson, 2001; Vega Rivera, Montaño, Rappole, & Cerda, 2009). An exception may be if vegetation growth was highly predictable over space and time, and measures of vegetation at different nests remained independent and did not converge. However, measuring vegetation at each nest on its fate date (depredation or hatching) may also be misleading, because vegetation at hatched nests developed over the full period of the nesting cycle, whereas vegetation at depredated nests was measured at an earlier point in the cycle. Critically, as several researchers have recently pointed out, measuring vegetation on the fate date can automatically lead to the erroneous conclusion that taller/denser vegetation is associated with higher nest survival when no effect is actually present (Borgmann & Conway, 2015; Gibson, Blomberg, & Sedinger, 2016; McConnell, Monroe, Burger, & Martin, 2017; Smith et al., 2018).

**Figure 2:**
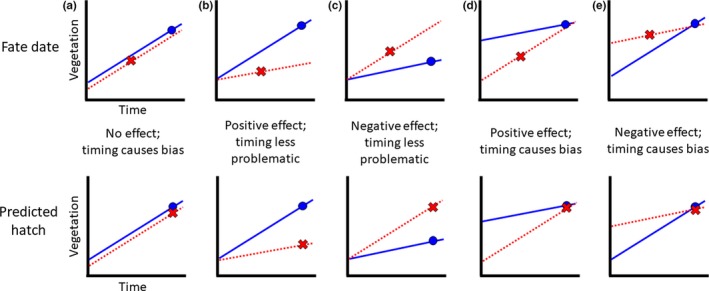
Depictions of potential bias in relation to timing of vegetation measurement, where upper plots show vegetation measured at fate date and lower plots show vegetation measured at actual or predicted hatch date. Blue lines represent hatched nests, and red lines represent depredated nests. Panel A shows no inherent effect of vegetation on nest survival (lines are identical [but slightly staggered for readability]). Panels B and D show inherent positive effects of vegetation on nest survival (hatched nests always found in denser vegetation). Panels C and E show inherent negative effects of vegetation on nest survival (depredated nests always found in denser vegetation). In panels A, D, and E, the timing of measurement can obscure the effect of vegetation on nest survival

Gibson et al. (2016) and McConnell et al. (2017) published concurrent and independent studies demonstrating that measuring vegetation at nests on their fate date leads to inflated estimates of the importance of vegetation on nest survival (Figure 2a). This bias is especially pronounced earlier in the season when vegetation is growing rapidly (McConnell et al., 2017). In simulation models where the true effect of vegetation density on nest survival was fixed (negative, neutral, or positive), parameter estimates obtained by measuring vegetation at nests on their fate dates were biased so high that even preprogrammed negative effects showed up as positive (Gibson et al., 2016). However, the magnitude of the bias uncovered in these simulation analyses is less pronounced in the empirical literature: Borgmann and Conway (2015) found that only 35% of passerine studies (*n* = 106) showed a positive effect of vegetation on nest survival when vegetation was measured near nest fate dates.

McConnell et al. (2017) advocated for measuring all nests at either their initiation date (often intractable, even with marked birds) or estimated hatch date. The latter method gives the vegetation at depredated nests more time to grow, and if growth is predictable, one can commensurately compare vegetation at hatched and (previously) depredated nests. Gibson et al. (2016) suggested standardizing vegetation by time of year by regressing vegetation on date and using the residuals as inputs into nest survival models. However, with these methods, systematic differences in vegetation growth between hatched and depredated nest sites could obscure the relationship (Figure 2). That notwithstanding, Smith et al. (2018) used a regression residuals approach to successfully uncover a bias in previously published models of greater sage‐grouse (*Centrocercus urophasianus*) nest survival, leading them to recommend revising management plans for this species of conservation concern.

The aforementioned studies do not consider an alternative time for measuring nest vegetation: when the nest is found by researchers. In many study systems, measuring nest vegetation at this time is avoided, so as to limit disturbance to the bird. However, in grassland‐nesting species such as dabbling ducks (*Anas, Mareca, *and *Spatula* spp.), nests are located most often by flushing birds from the nest—which makes it an opportune time to measure nest vegetation. This accomplishes a similar objective as measuring vegetation on estimated fate date (as advocated by McConnell et al. (2017)), which is to dissociate the timing of measurement from the process of interest (predation). However, unlike measuring every nest at the end of the expected nesting cycle, here each nest is measured on a random day of the year at a random time during the nesting cycle. In this analysis, we used data from ~2,800 duck nests where we measured vegetation at the time of nest discovery and again at the time of nest fate to address: (a) evidence of bias in either estimate, (b) how statistical correction factors (sensu Gibson et al., 2016) compare to raw vegetation estimates, and (c) whether there are systematic differences in vegetation growth between hatched and depredated nests that could further obscure the relationship. We also reviewed the literature to provide an overview of which methods are most often used in research on upland‐nesting ducks, one of the most intensively studied systems in North America.

## 2 METHODS

### 2.1 Literature review

We sampled the literature on duck nesting ecology to determine when vegetation at the nest is most often measured by researchers. North American ducks are some of the most intensively studied birds in the world (U.S. Fish & Wildlife Service, 2012), and a full review of all nesting studies is beyond the scope of this paper (e.g., a simple search on the Web of Science database for “duck nest survival” returned 363 results). Instead, we based our review around the Robel, Briggs, Dayton, and Hulbert (1970) study which described a simple method for estimating vegetation density at upland bird nests. While unique study‐specific methods for measuring vegetation occur in the duck nesting literature (e.g., Crabtree, Broome, and Wolfe (1989)), Robel pole measurements are commonly used across geographies and contexts, and provide a convenient method to identify literature on duck nest vegetation. We evaluated the posterior citation tree for the Robel et al. (1970) paper using the Web of Science database (June 2018), which identified 514 studies that cited the original paper. We searched within these 514 studies using the terms “duck nest” which yielded 38 results, of which 36 were research studies on nesting waterfowl.

### 2.2 Empirical data

Our duck nesting data were collected in 2016 and 2017 from northwestern North Dakota, USA (Ward, Mountrail, Burke, and Divide counties). We selected 10.4 km^2^ sites spread across the geography (*n* = 37 in 2017, *n* = 34 in 2018), requiring them to contain >47% perennial cover and >100 wetland basins to ensure an adequate sample of breeding ducks (Stephens, Rotella, Lindberg, Taper, & Ringelman, 2005). At each site, we attempted to search for nests on at least two replicate parcels of upland nesting cover 32.4 ha in size. We conducted morning nest searches (Gloutney, Clark, & Afton, 1993) approximately every 3 weeks between late April and early July to ensure we found early‐ and late‐nesting duck species. We searched for duck nests using a chain drag (Klett, Duebbert, Faanes, & Higgins, 1986). Briefly, we strung a ~60 m steel chain between two all‐terrain vehicles and drove them parallel through upland nesting habitat; dragging the chain across the top of the vegetation caused the female to flush from the nest. After the nest was found, we flagged vegetation with red duct tape 4 m north of the nest bowl to minimize detection by avian predators (Hein & Hein, 1996).

At the nest, we recorded the number of eggs and the incubation stage determined by candling (Weller, 1956), which sum to the age of the nest. On the day, the nest was found (“found date”), we quantified the density of vegetation around the nest using a Robel pole (Robel et al., 1970)—our closest measure of vegetation at the time of nest‐site selection. We placed a 1.5 m measuring pole at the nest bowl and recorded the lowest measurement marking visible from 3.7 m away as viewed from 0.9 m above the ground. Markings were delineated in inches to provide commensurate data with previous research at our sites; in this paper, we did not convert these measurements to metric (cm or dm) because that would misrepresent the precision we empirically achieved in the field. Measurements were taken from each of the four cardinal directions and averaged. Robel measurements convey information about the density of vegetation at the nest (Robel et al., 1970) and provide a useful index of visual and potentially olfactory concealment from common nest predators. We recorded nest locations with a GPS receiver and revisited them on foot every 5–7 days until the nest either hatched or failed. During the final visit to the nest (after hatching or depredation; “fate date”), we recorded a second set of Robel measurements—which we thought at the time might be related to nest fate, but perhaps not in light of the bias revealed in studies by Borgmann and Conway (2015), Gibson et al. (2016), and McConnell et al. (2017). We did not revisit depredated nests at predicted fate date, simply because the logistics of making date‐specific visits to 1,717 nests spread across a 40,000 km^2^ geography were intractable. A nest was considered successful if ≥1 egg hatched. We assumed nests found abandoned on the first recheck were influenced by investigator disturbance and were censored from our analyses. All fieldwork was carried out under Louisiana State University Institutional Animal Care and Use permit #15‐017 and North Dakota collecting permit GNF03793985.

**Figure 3:**
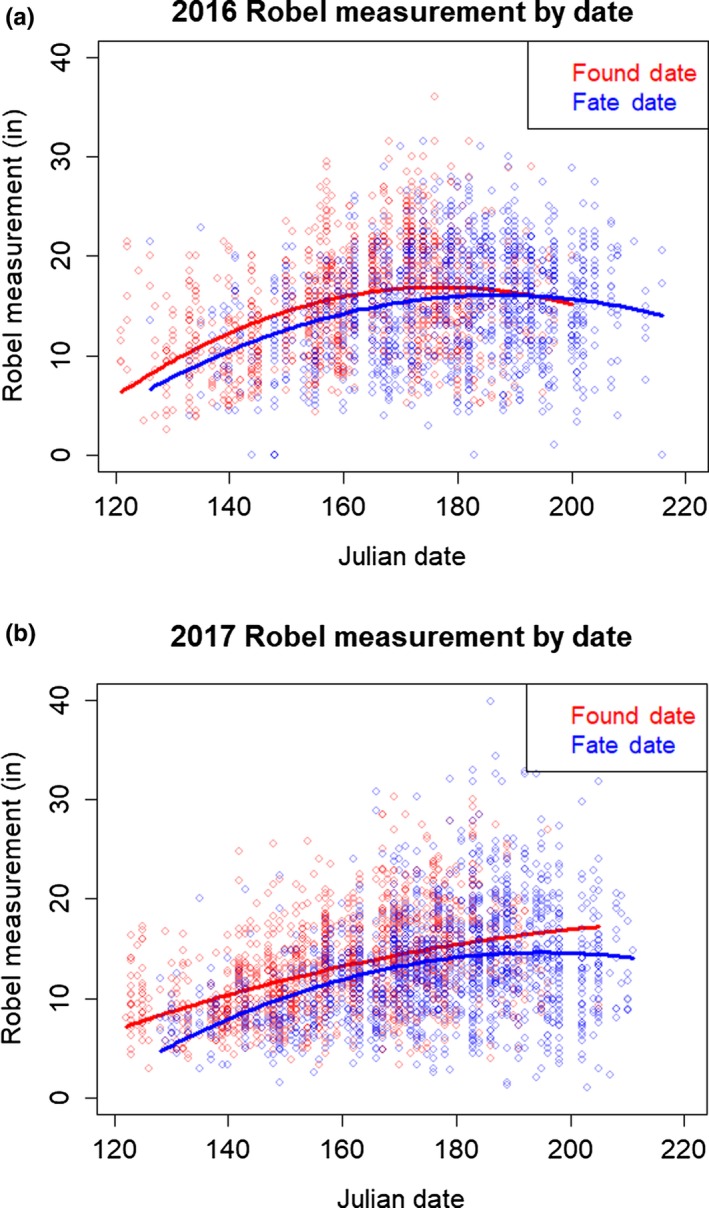
Raw data and best‐fit quadratic models for Robel vegetation density data collected at duck nests in northwestern North Dakota, USA, in 2016 and 2017. Vegetation data were collected on the date the nest was discovered (“found date”), and at the last visit to the nest when the attempt found terminated (“fate date”)

We conducted nest survival analyses (Dinsmore, White, & Knopf, 2002) in program MARK (White & Burnham, 1999) accessed through the RMark package (Laake, 2013). We developed a preliminary model set that included variables for species, age of the nest when found, and initiation date, all of which are known to have an influence on nest survival (Ringelman, Walker, Ringelman, & Stephens, 2018). To determine whether including vegetation metrics improved fit of the top model, we sequentially tested the addition of the two raw Robel measurements, empirically collected in the field at found date and fate date. Following Gibson et al. (2016), we also created date‐corrected versions of these raw Robel measurements for inclusion in survival models. To accomplish this, we fit linear and quadratic models to Robel measurements collected at both found date and fate date; slopes and intercepts of vegetation growth could plausibly vary between years, so we modeled 2016 and 2017 separately. In all four cases, quadratic models provided the best fit to the data (AICc weights >88% for quadratic models) (Figure 3). We used the residuals from the best‐fit quadratic model as our estimates of date‐corrected vegetation measurements and sequentially added them to our nest survival model set. We evaluated candidate models using AICc scores (Burnham & Anderson, 2002) and compared parameter estimates and daily survival rates estimated from models containing each type of vegetation measurement.

An alternative method for diagnosing potential bias caused by differential timing of measurement of hatched and depredated nests is to restrict comparisons to only those nests that had contemporaneous fate dates. We created subsets of nests by sorting them into 10‐day bins based on their fate dates (e.g., a bin of nests fated between ordinal date 151 and 160) and reran our survival analyses using raw Robel measurements collected at fate date (which should now be relatively unbiased because we are comparing nests fated around the same time). We selected 10 days as a compromise between restricting the vegetation growth window and achieving adequate sample sizes. We required each bin to contain >50 nests for survival analyses and discarded the earliest bins of nests (ordinal dates <150), which were depauperate in hatched nests.

Finally, we were interested in how vegetation changes through time at each nest: specifically, if there was a consistent difference in vegetation growth between hatched and depredated nests. We parsed the nest data by fate and used paired *t*‐tests of Robel measurements (at found date and fate date) to determine how vegetation changed at each nest over the course of the monitoring period. For comparison with previous work and to provide a frame of reference for our other analyses, we also calculated the raw difference in Robel measurement between the fate date and found date for each nest and used this as yet another metric of vegetation in a final set of survival analyses. All statistics were conducted in program R (version 3.5.0), and we report means ± standard errors unless otherwise noted.

## 3 RESULTS

### 3.1 Literature review

We reviewed 36 studies that used Robel vegetation measurements to make inferences about duck breeding ecology (Table 1). The most common practice (*n* = 18 studies) was to measure vegetation at several points in a field at a fixed point in time (e.g., typically on a specific calendar date) and use that to draw inferences about how a particular management regime influenced vegetation at scales larger than individual nest sites; in those cases, the relationship between Robel measurements and nest survival was not explicitly tested. Several studies using artificial nests (*n* = 6) measured vegetation at a fixed point in time and found either a positive effect (*n* = 3) or no effect of (*n* = 3) vegetation on nest survival, but artificial nests may not reflect predation rates at natural nests (Butler & Rotella, 1998). When timing of measurement was not fixed, it most often occurred when the nest was found (*n* = 14), rather than at fate date (*n* = 2), and positive, neutral, and negative effects on nest survival were all observed (Table 1). Thompson, Arnold, and Vacek (2012) found a positive effect when they accounted for bias by using a vegetation*initiation date interaction term, and Koons and Rotella (2003) were the only researchers to measure nest vegetation at the estimated hatch date for all nests (sensu McConnell et al., 2017) and found no effect of vegetation on survival.

**Table 1:** Review of studies that use Robel vegetation measurements to study duck breeding ecology. Studies that do not explicitly relate vegetation to nest success are noted by “N/A.”

Timing of measurement	Site measured	Effect on nest success	Study
Fixed date	Field	N/A	(Luttschwager, Higgins, & Jenks, 1994)
Fixed date	Field	N/A	(Greenwood, Pietruszewski, & Crawford, 1998)
Fixed date	Field	N/A	(Lapointe, Giroux, Belanger, & Filion, 2000)
Fixed date	Field	N/A	(Piest & Sowls, 1985)
Fixed date	Field	N/A	(Shaffer et al., 2006)
Fixed date	Field	N/A	(West & Messmer, 2006)
Fixed date	Field	N/A	(Bélanger & Picard, 1999)
Fixed date	Field	N/A	(Zicus, Rave, Das, Riggs, & Buitenwerf, 2006)
Fixed date	Field	N/A	(Carroll, Arnold, & Beam, 2007)
Fixed date	Field	N/A	(Kantrud, 1993)
Fixed date	Field	N/A	(Haffele, Eichholz, & Dixon, 2013)
Fixed date	Field	N/A	(Devries & Armstrong, 2011)
Fixed date	Field	Positive effect—artificial nests	(Butler & Rotella, 1998)
		No effect—natural nests	
Fixed date	Field		(Gabrey, Wilson, & Afton, 2002)
Fixed date	Nest	N/A—artificial nests	
Fixed date	Nest	No effect—artificial nests	(Esler & Grand, 1993)
Fixed date	Nest	No effect—artificial nests	(Olson & Rohwer, 1998)
Fixed date	Nest	Positive effect—artificial nests	(Vander Lee, Lutz, Hansen, & Mathews, 1999)
Fixed date	Nest	Positive effect—artificial nests	(Clawson & Rotella, 1998)
Fixed date	Field	N/A	(Kruse & Bowen, 1996)
Found date	Nest		
Fixed date	Field	N/A	(Duebbert & Kantrud, 1987)
Found date	Nest		
Fixed date	Field		(Warren, Rotella, & Thompson, 2008)
Found date (year 1);every week through fate (year 2)	Nest	Positive effect across most field vegetation regimes	
Fixed date	Field		(Ackerman, 2002)
Found date	Nest	No effect	
Fixed (twice)	Field		(Bloom, Howerter, Emery, & Armstrong, 2013)
Found date	Nest	Positive	
Found date	Field		(Durham & Afton, 2003)
Found date	Nest	Positive effect	
Found date	Nest	N/A	(Loos & Rohwer, 2002)
Found date	Nest	N/A	(Ringelman et al., 2017)
Found date	Nest	N/A	(Stephens et al., 2005)
Found date	Nest	No effect	(Péron, Walker, Rotella, Hines, & Nichols, 2014)
Found date	Nest	No effect	(Ringelman, Eadie, & Ackerman, 2014)
Found date	Nest	Positive effect	(Raquel, Ringelman, Ackerman, & Eadie, 2015)
Found date	Nest	Positive effect	(Ringelman et al., 2018)
Found date	Nest	Negative effect	(Skone, Rotella, & Walker, 2016)
Fate date	Field		(Varner, Bielefeld, & Hepp, 2013)
Fate date	Nest	No effect	
Fate date	Nest	N/A	(Hoekman, Ball, & Fondell, 2002)
Fate date × initiation date interaction term	Nest	Positive effect	(Thompson et al., 2012)
Estimated fate	Nest	No effect on natural nests	(Koons & Rotella, 2003)

### 3.2 Empirical data

In 2016 and 2017, we searched for duck nests on a total of 6,063 ha of upland habitat in North Dakota and located 2,993 nests, of which 2,794 were suitable for analysis. We located nests of eight different species: gadwall (*Mareca strepera*) were the most numerous (*n* = 769), followed by blue‐winged teal (*Spatula discors*; *n* = 759), mallard (*Anas platyrhychos*; *n* = 578), and northern shoveler (*Spatula clypeata*; *n* = 237). We found less than 200 nests of northern pintail (*Anas acuta*), lesser scaup (*Aythya affinis*), American wigeon (*Mareca americana*), and green‐winged teal (*Anas crecca*). All nests were located in qualitatively similar upland habitat, and so we included all species in our analysis.

We first constructed a set of basic nest survival models, which included combinations of variables that are well‐known to influence nest fate: species, age of the nest when found, and initiation date. Our top model contained all three variables and was the only competitive model (second‐ranked model ΔAICc > 6). To determine whether vegetation was a meaningful predictor of nest survival, we sequentially added the four metrics of vegetation to the top model and also tested them as stand‐alone models. Adding any type of vegetation metric improved model fit >4 AICc over the baseline survival model (Table 2). The univariate model of raw vegetation measurement at fate date was an anomalously powerful predictor of nest survival—evidently even more important than our baseline model of species, age of the nest, and initiation date (Table 2).

**Table 2:** Model selection results from analyses of duck nest survival. Including any of the four nest vegetation metrics improved model fit over the baseline survival model (Species + Age of nest when found + Nest initiation date). “Found date” and “fate date” refer to Robel measurements taken when the nest was first located and last visited. Residuals were taken from the best‐fit year‐specific quadratic regression model of Robel measurement on nest initiation date

Parameters	*K*	Log likelihood	AICc	ΔAICc
Baseline + raw fate date	11	−3493.3	7008.7	0.0
Raw fate date	2	−3517.5	7039.0	30.3
Baseline + residual found date	11	−3517.2	7056.4	47.7
Baseline + raw found date	11	−3518.3	7058.7	50.0
Baseline + residual fate date	11	−3518.5	7058.9	50.3
Baseline	10	−3521.4	7062.7	54.1
Residual found date	2	−3537.3	7078.5	69.9
Residual fate date	2	−3538.2	7080.5	71.8
Raw found date	2	−3539.2	7082.4	73.8
[null]	1	−3540.9	7083.8	75.2

We then examined a simplified model set containing only the four single‐parameter models of each vegetation metric to avoid confounding factors (e.g., residual vegetation metrics and initiation date) and for simplicity of interpretation (pooling species). All four single‐variable vegetation models estimated a similar daily survival rate for nests (range: 0.9492–0.9494). However, the raw fate date model estimated the effect of vegetation (*β* = 0.032 ± 0.005) as twice that of the residual found date model (*β* = 0.016 ± 0.006), and nearly three times that as the residual fate date model (*β* = 0.011 ± 0.005) and raw found date model (*β* = 0.009 ± 0.005).

**Figure 4:**
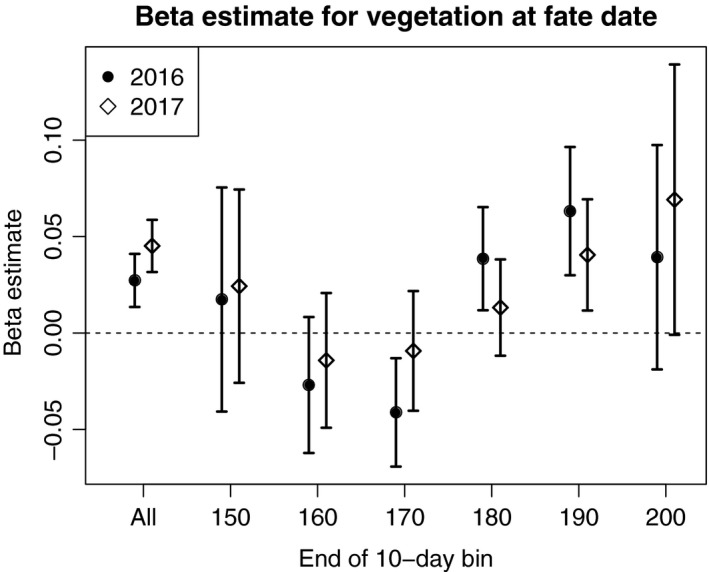
Beta estimates and 95% confidence intervals for the effect of vegetation (measured at fate date) on duck nest survival. The effect is reliably positive in 2016 and 2017 when analyzing data from all nests simultaneously, but effects are less pronounced when only examining hatched and depredated nests that were fated within the same 10‐day window (ordinal date bins shown for each year)

In our next analysis, we binned nests that were concurrently terminated within a 10‐day date range (parsed by year), which should limit potential bias in the timing of measurement between depredated and hatched nests. For each date‐year bin (*n* = 12), we ran simple survival analyses with one parameter—raw vegetation measurement at fate date—and compared coefficient estimates from each bin to the global estimate which pooled all nests (described above). Pooling all nests, coefficient estimates for the effect of vegetation on nest survival were reliably positive (Figure 4). In contrast, 95% confidence intervals bounded zero for 8 out of 12 date bins, and beta estimates were both positive and negative (Figure 4). Interestingly, in both 2016 and 2017 there were trends for vegetation at the nest to have a negative effect on survival during the first 20 days in June, but a positive effect later in the season. Early season effects were not evaluated because successful nests were rare at that time of year (because they take ~35 days to hatch).

Interestingly, despite significant positive trends in vegetation density across a season (Figure 3), paired tests at the nests showed no significant pattern of vegetation growth at individual nests (*µ*_found_ = 14.16, *µ*_fate_ = 14.11, *p* = 0.45). This non‐significance appears to stem from the fact that vegetation at successful nests becomes significantly denser over the course of a nesting attempt (*µ*_found_ = 14.71, *µ*_fate_ = 15.10, *p* < 0.01), while vegetation at depredated nests actually becomes less dense (*µ*_found_ = 13.81, *µ*_fate_ = 13.48, *p* < 0.01). From these averages, biological effect sizes seem small (i.e., less than half an inch on a Robel pole), but differences were larger within individual nests. When the difference in Robel measurements between found date and fate date is included as a candidate model in our nest survival analyses, it receives overwhelming support (AIC = 7029.2, Table 2), improving on the raw fate date model by more than 9 AIC units.

## 4 DISCUSSION

Waterfowl researchers have been studying the influence of vegetation on nest survival for more than three decades. Unlike many studies which measure nest vegetation when the nest becomes inactive (depredated or hatched), waterfowl studies tend to measure vegetation at the time the nest was found, potentially avoiding biased estimates of the effect of vegetation on nest fate (Gibson et al., 2016; McConnell et al., 2017). We found only one study in our sample (Koons & Rotella, 2003) that measured vegetation at depredated nests on the date of estimated hatch as recommended by McConnell et al. (2017). One plausible explanation is that given the expansive geography and large sample sizes achieved by many waterfowl studies, it simply may be logistically intractable to revisit depredated nests after they are terminated to measure vegetation.

Using empirical data from ~2,800 nests, we found that each of our four vegetation metrics significantly influenced nest survival. In support of hypotheses proposed by Gibson et al. (2016) and McConnell et al. (2017), we found that our raw measurements of vegetation collected at the time of nest fate were an anomalously strong predictor of nest survival. Correcting raw vegetation metrics using regression residuals as suggested by Gibson et al. (2016) reduced the effect size of vegetation on nest survival and was commensurate with raw vegetation metrics collected when the nest was found. Taken in combination with the literature, our analysis suggests that correcting vegetation metrics using regression residuals or using raw vegetation measurements when the nest was found probably both yield less biased estimates of the influence of vegetation on nest survival than using raw vegetation data collected on nest fate date.

Measuring vegetation at nests that are terminated (either hatch or fail) around the same time has the potential to reduce bias, but rests on the assumption that vegetation does not appreciably change across each observation window. We selected 10‐day bins in an effort to meet this assumption while achieving sufficient sample sizes (we required >50 nests in each bin). Looking across all nests in a season (no date binning), the effect of fate date vegetation was reliably positive and possibly biased (Figure 4). However, that positive effect—similar in magnitude but with greater uncertainty—was also observed in some date bins which should be relatively free from any bias relating to vegetation growth. So it seems plausible that for some nests, vegetation as measured at fate date is an appropriate and meaningful predictor of nest survival. Perhaps even more interesting was the apparent negative effect of vegetation on nest survival observed during the first 20 days of June. We suspect that this was driven by between‐species variation in the timing of nesting, nest vegetation preferences, and nest survival rates. Specifically, the most common species being monitored at that time was mallard, which nests in taller vegetation (Clark & Shutler, 1999) and has consistently lower survival rates (Stephens et al., 2005) than later nesting (Raquel et al., 2016) blue‐winged teal and gadwall. Additionally, concealment in vegetation may become a more important determinant of nest survival later in the season as predators may learn to cue on duck nests (Larivière & Messier, 1998; Ringelman et al., 2018).

Our final set of analyses uncovered that vegetation at hatched nests increased in density over the course of the nesting cycle, whereas vegetation at depredated nests significantly decreased in density. In essence, this situation is an extreme case of that represented in Figure 2e, except the slope of vegetation growth at the depredated nest is negative and the intercept is shifted below the intercept for hatched nests. In this case, measurement bias increases later in the nesting cycle because the two lines diverge; measuring at estimated hatch date would be the most biased scenario here, and residual‐based methods would converge. Why does vegetation at depredated nests become less dense between the found date and the fate date? One possible explanation is that disturbance of vegetation by a predator causes average Robel readings to be lower at our fate date measurement. However, in our experience, wanton destruction of vegetation at duck nests by predators is uncommon and is probably an insufficient explanation for vegetation differences across 1,717 depredated duck nests. Instead, there are several ecological explanations for why vegetation measurement may decrease at some nest sites. For example, in our prairie system, insufficient moisture, senescence, disturbance from humans and livestock, and grass being physically being beaten down by rain and wind are all plausible explanations for declining vegetation measurements at nest sites—and those then become the sites that are more likely to be discovered by predators. When we included the difference in Robel fate date measurements in our nest survival model set, it clearly provided the best fit to the data. On the one hand, this makes sense: if birds choose nest sites adaptively, that is, select a site that provides sufficient concealment, then how well that concealment is maintained over the course of the nesting cycle could be a critical determinant of predation risk. On the other hand, given patterns of diverging vegetation growth, comparing differences in Robel readings between hatched and depredated nests may be even more biased that scenarios modeled by Gibson et al. (2016) and McConnell et al. (2017).

The study presented here adds to a growing literature that emphasizes the importance of plant phenology in researching nest survival. Our nest survival models, taken in combination with a review of the duck nesting literature, indicate that the majority of waterfowl studies have collected vegetation data using relatively less biased methods (date when found). Results of previous research have been mixed: Some, but not all studies have found that higher vegetation density at the nest is associated with higher nest survival. We uncovered that positive relationship in our data, and given the diversity of analyses presented here, we are confident that a true effect exists. Interestingly, because of the late‐season divergence in vegetation between hatched and depredated nests, using vegetation data collected on the date when the nest was found would likely underestimate any effect present; this may explain the lack of a strong pattern in the literature. We were unable to determine whether the difference in vegetation growth patterns per se between hatched and depredated nests is an important biological explanation for predation risk. Experimental manipulation of nest vegetation would help bypass this observational bias and would be a fruitful direction for future research. In the meantime, collecting additional vegetation data at different points in the nesting cycle (e.g., at every nest visit and at predicted nest fate) would help shed light on the systematic and directional changes in vegetation revealed here.

## AUTHOR CONTRIBUTION

K.M.R. and C.G.S. formulated the questions; K.M.R. acquired the funding; C.G.S. supervised data collection in the field; K.M.R. analyzed the data; and K.M.R. wrote the manuscript with the help of edits from C.G.S.

## Data Availability

Empirical nesting data presented in this paper are available at Dryad: https://doi.org/10.5061/dryad.4j36s87.
